# Supplementary figures and images for: Antibacterial activity of essential oils from Ethiopian thyme (Thymus serrulatus and Thymus schimperi) against tooth decay bacteria
Source: PLoS One. 2020 Oct 9;15(10):e0239775. doi: 10.1371/journal.pone.0239775 (PMC7546913; doi:10.1371/journal.pone.0239775)

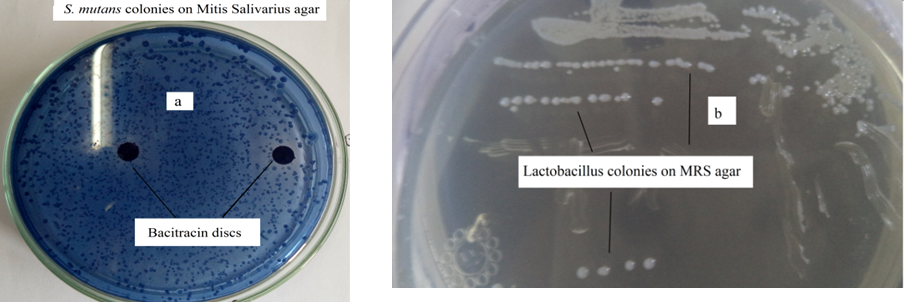


a

**S1_Fig. tif** Isolation of *S. mutans* (a) and *Lactobacillus* (b) colonies on MS and MRS agars respectively

Supplement: S1 Fig — Isolation of S. mutans (a) and Lactobacillus (b) colonies on MS and MRS agars respectively. (DOCX) [file pone.0239775.s001.docx]

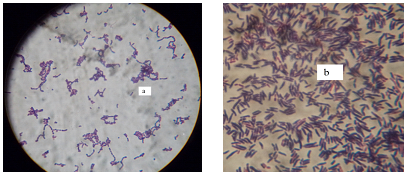


**S2_Fig.tif.** Results for Gram staining experiments on *S. mutans* (a) and *Lactobacillus* (b)

Supplement: S2 Fig — Results for Gram staining experiments on S. mutans (a) and Lactobacillus (b). (DOCX) [file pone.0239775.s002.docx]

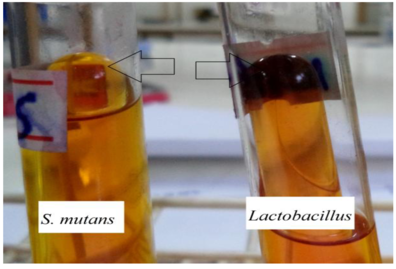


S4_Fig.tif. Aerobic growth test result of *S. mutans* and *Lactobacillus*

Supplement: S4 Fig — (DOCX) [file pone.0239775.s004.docx]

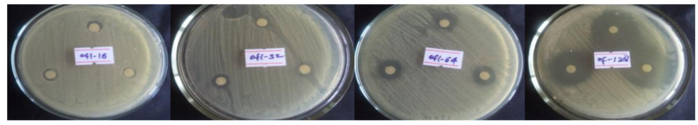


S5_Fig.tif. Inhibition of Lactobacillus by Ofl EO (doses: 16μl/mL, 32μl/mL, 64μl/mL, and 128μl/mL respectively)

Supplement: S5 Fig — (DOCX) [file pone.0239775.s005.docx]

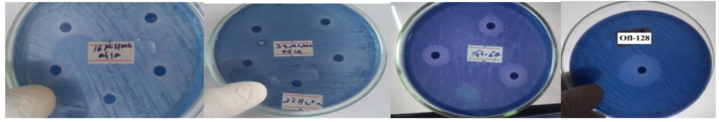


S6_Fig.tif. Inhibition of *S. mutans* by Ofl EO (doses: 16μl/mL, 32μl/mL, 64μl/mL, and 128μl/mL respectively)

Supplement: S6 Fig — (DOCX) [file pone.0239775.s006.docx]

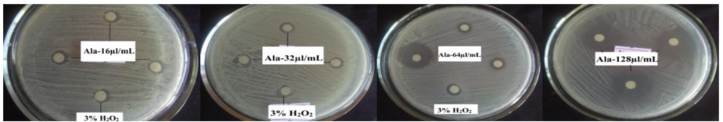


S7_Fig.tif. Inhibition of *Lactobacillus* by Ala EO (doses: 16μl/mL, 32μl/mL, 64μl/mL, and 128μl/mL respectively)

Supplement: S7 Fig — (DOCX) [file pone.0239775.s007.docx]

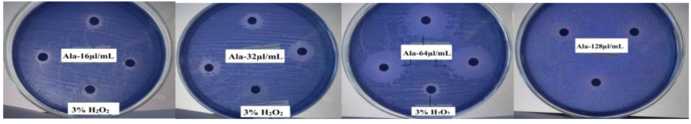


S8_Fig.tif. Inhibition of *S. mutans* by Ala EO (doses: 16μl/mL, 32μl/mL, 64μl/mL, and 128μl/mL respectively)

Supplement: S8 Fig — (DOCX) [file pone.0239775.s008.docx]

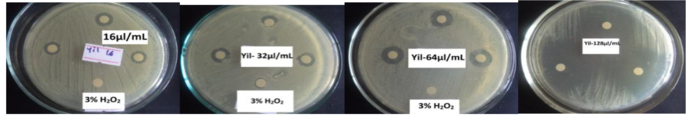


S9_Fig.tif. Inhibition of *Lactobacillus* by Yil EO (doses: 16μl/mL, 32μl/mL, 64μl/mL, and 128μl/mL respectively)

Supplement: S9 Fig — (DOCX) [file pone.0239775.s009.docx]

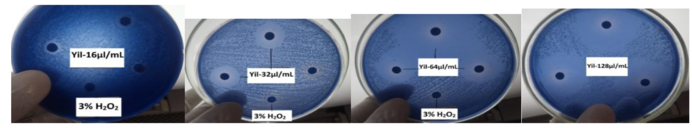


S10_Fig.tif. Inhibition of *S. mutans* by Yil EO (doses: 16μl/mL, 32μl/mL, 64μl/mL, and 128μl/mL respectively)

Supplement: S10 Fig — (DOCX) [file pone.0239775.s010.docx]

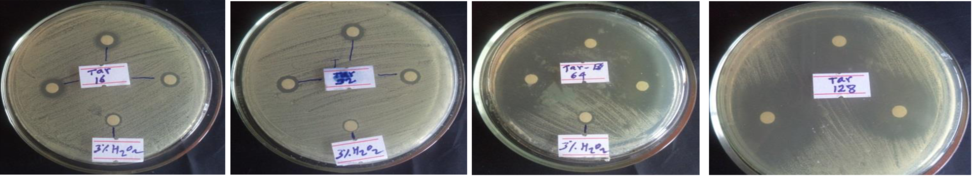


S11_Fig.tif. Inhibition of *Lactobacillus* by Tar EO (doses: 16μl/mL, 32μl/mL, 64μl/mL, and 128μl/mL respectively)

Supplement: S11 Fig — (DOCX) [file pone.0239775.s011.docx]

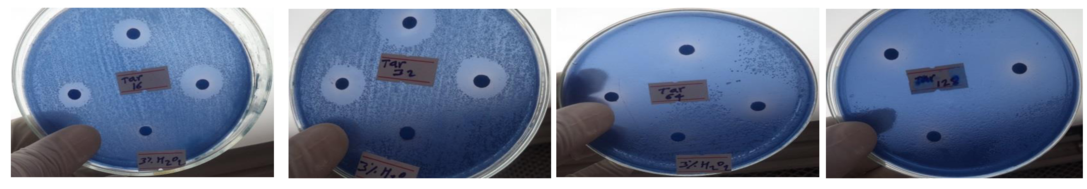
S12_Fig.tif. Inhibition of *S. mutans* by Tar EO (doses: 16μl/mL, 32μl/mL, 64μl/mL, and 128μl/mL respectively)

Supplement: S12 Fig — (DOCX) [file pone.0239775.s012.docx]

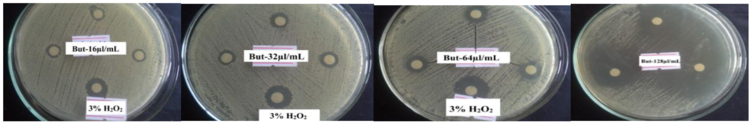


S13_Fig.tif. Inhibition of *Lactobacillus* by Buta EO (doses: 16μl/mL, 32μl/mL, 64μl/mL, and 128μl/mL respectively)

Supplement: S13 Fig — (DOCX) [file pone.0239775.s013.docx]

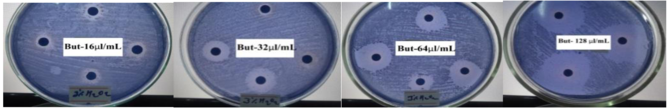


S14_Fig.tif. Inhibition of *S. mutans* by Buta EO (doses: 16μl/mL, 32μl/mL, 64μl/mL, and 128μl/mL respectively)

Supplement: S14 Fig — (DOCX) [file pone.0239775.s014.docx]

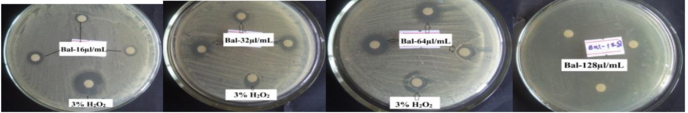


S15_Fig.tif. Inhibition of *Lactobacillus* by Bal EO (doses: 16μl/mL, 32μl/mL, 64μl/mL, and 128μl/mL respectively)

Supplement: S15 Fig — (DOCX) [file pone.0239775.s015.docx]

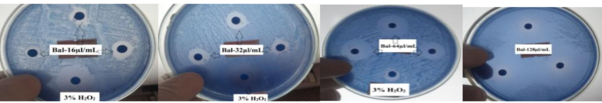


S16_Fig.tif. Inhibition of *S. mutans* by Bal EO (doses: 16μl/mL, 32μl/mL, 64μl/mL, and 128μl/mL respectively)

Supplement: S16 Fig — (DOCX) [file pone.0239775.s016.docx]

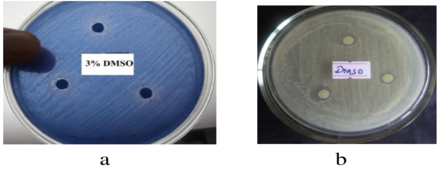


S17_Fig.tif. Inhibition of *S. mutans* (a) and *Lactobacillus* (b) by 3% DMSO

Supplement: S17 Fig — Inhibition of S. mutans (a) and Lactobacillus (b) by 3% DMSO. (DOCX) [file pone.0239775.s017.docx]

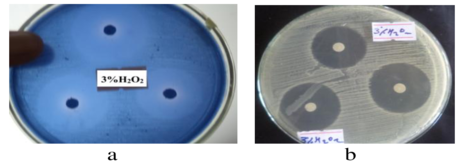


S18_Fig.tif. Inhibition of *S. mutans* (a) and *Lactobacillus* (b) by 3% H2O2

Supplement: S18 Fig — Inhibition of S. mutans (a) and Lactobacillus (b) by 3% H2O2. (DOCX) [file pone.0239775.s018.docx]

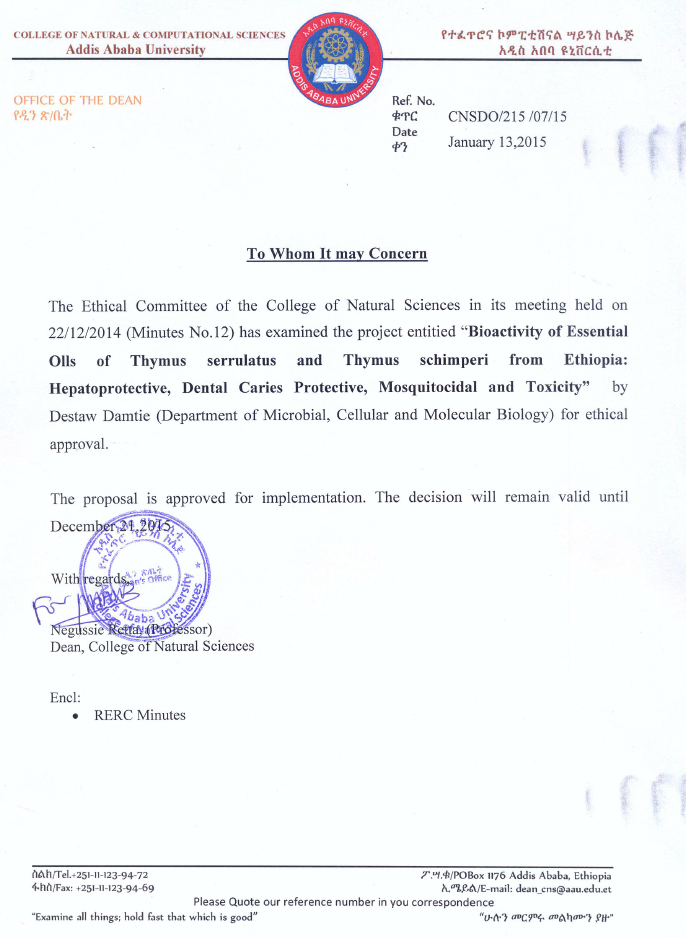

Supplement: S1 File — (DOCX) [file pone.0239775.s019.docx]
