# Supplementary material for: Antibacterial activity of essential oils from Ethiopian thyme (Thymus serrulatus and Thymus schimperi) against tooth decay bacteria
Source: PLoS One. 2020 Oct 9;15(10):e0239775. doi: 10.1371/journal.pone.0239775 (PMC7546913; doi:10.1371/journal.pone.0239775)
Supplement: S3 Fig — Sugar hydrolysis and arginine hydrolysis test results of S. mutans (a) and Lactobacillus (b). (DOCX) [file pone.0239775.s003.docx]

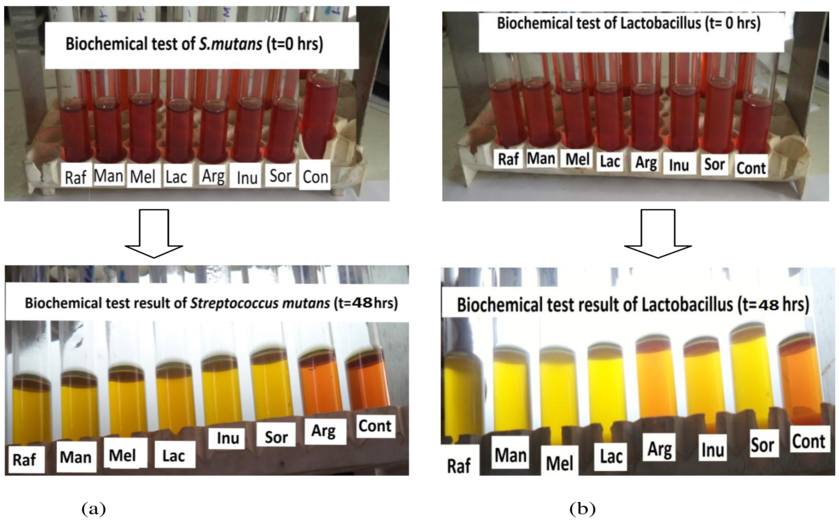


b

S3_Fig.tif. Sugar hydrolysis and arginine hydrolysis test results of *S. mutans* (a) and *Lactobacillus* (b)
