# Supplementary material for: Antibacterial activity of essential oils from Ethiopian thyme (Thymus serrulatus and Thymus schimperi) against tooth decay bacteria
Source: PLoS One. 2020 Oct 9;15(10):e0239775. doi: 10.1371/journal.pone.0239775 (PMC7546913; doi:10.1371/journal.pone.0239775)
Supplement: Attachment — Submitted filename: Response.docx [file pone.0239775.s020.docx]

PONE-D-20-17109

**Response to Reviewers**

**Dear Editor- in-Chief,**

Thank you for giving us the opportunity to submit a revised draft of the manuscript “Antibacterial activity of essential oils from Ethiopian thyme (Thymus serrulatus and Thymus schimperi) against tooth decay bacteria” for publication in PLOS ONE. We appreciate the time and effort that you and the reviewers dedicated to providing feedback on our manuscript and are grateful for the insightful comments on and valuable improvements to our paper. We have incorporated most of the suggestions made by the reviewers. Those changes are highlighted within the manuscript. Please see below, in blue, for a point-by-point response to the reviewers’ comments and concerns. All page numbers refer to the revised manuscript file with tracked changes.

Kindly,

Desta Damtie

**Comment 1:** Please ensure that your manuscript meets PLOS ONE's style requirements, including those for file naming. The PLOS ONE style templates can be found at

<https://journals.plos.org/plosone/s/file?id=wjVg/PLOSOne_formatting_sample_main_body.pdf> and

<https://journals.plos.org/plosone/s/file?id=ba62/PLOSOne_formatting_sample_title_authors_affiliations.pdf>

Response 1: Gone through and corrected

**Comment 2:** In your Methods section, please provide additional location information of the collection sites, including geographic coordinates for the data set if available.

Response 2: Added (Lines 84-87)

**Comment 3:** In your Methods section, please provide additional information regarding the permits you obtained for the work. Please ensure you have included the full name of the authority that approved the collection site access and, if no permits were required, a brief statement explaining why.

Response 3: These plants are wild plants which were found in communal lands of the study sites. Besides, these plants are not endangered or protected species. Therefore, specific permission was not required for the collection and investigation of the mentioned plants. (Lines 86-88)

**Comment 4:** In your Methods section, please provide additional information about the participant recruitment method and the demographic details of your participants. Please ensure you have provided sufficient details to replicate the analyses such as: a) the recruitment date range (month and year), b) a description of any inclusion/exclusion criteria that were applied to participant recruitment, c) a table of relevant demographic details, d) a statement as to whether your sample can be considered representative of a larger population, e) a description of how participants were recruited, and f) descriptions of where participants were recruited and where the research took place.

Response 4: The participants served only as sources of bacteria. The study was conducted on bacteria. The findings are generalized to bacteria not to sources of bacteria. What was required is Ethical clearance and it is attached with the supplementary materials.

**Comment 5:** Thank you for stating the following in the Funding Section of your manuscript:

[The Ministry of Education of Ethiopia, Addis Ababa University, and Bahirdar University funded this research work.]

We note that you have provided funding information that is not currently declared in your Funding Statement. However, funding information should not appear in the Acknowledgments section or other areas of your manuscript. We will only publish funding information present in the Funding Statement section of the online submission form.

Please remove any funding-related text from the manuscript and let us know how you would like to update your Funding Statement. Currently, your Funding Statement reads as follows:

 [No]

Response 5: Edited as “The authors received no specific funding for this work.

“ (Line 382)

# **Reviewer 1**

## **Comment 1:**

- The introduction is too short and needs implementing. The other activities of Thymus and EOs in general could be discussed: see for example “Mediterranean essential oils as precious matrix components and active T ingredients of lipid nanoparticles, Int. J. Pharm. 548 (2018) 217–226”.

Response 1: Introduction is lengthened by addition of further information

- Lines 35-60.
- Lines 64-67

## **Comment 2:**

- Section “Plant material collection and identification”: thymus collected in Ofla is not reported, while it is mentioned in the abstract.

## **Response 2:**

- Corrected (Line 82)

## **Comment 3:**

- Line 79: even though a reference was provided, a brief description of the procedure followed to collect saliva should be provided for the benefit of the reader.

## **Response 3:**

- Corrected (Lines 109-117)

# **Reviewer 2**

## Comment 1

- Essential oil production is not appropriate term. Its better to use professional terms.

## Response 1

- Corrected as “Preparation of plant materials and extraction of essential oils” (Line 92)

## Comment 2

- "Aerial parts of T. serrulatus and T. schimperi collected from the wild ….. ". This sentence needs edition.

## Response 2

- Corrected as “The collected T. serrulatus and T. schimperi plants were shade dried and milled into powders.” (Lines 93-94)

## Comment 3

- Please describe the process of dilution of essential oils and pouring them in the agar

## Response 3

- Described in Lines 150-152

## Comment 4

- "They too were not able to release gas from H2O2"

## Response 4

- Modified as “They too were catalase-negative” (Line 190)

## Comment 5

- "The EOs doses ranging from 16 μl/mL to 128 μl/mL of Ala too showed" needs edition.

## Response 5

- Modified as “In the same way, the EOs doses ranging from 16 μl/mL to 128 μl/mL of Ala showed considerable inhibition to this bacterium even though their zones of inhibition were less than that of the 3% H2O2” (Lines 212-214)

## Comment 6

- Its better to define exactly the terms in tables such as : Yil , Tar,Ala ,Buta and ….

## Response 6

- Corrected (Lines 231 & 277)

## Comment 7

- "The EOs of thyme in the present study contained components with high antibacterial activities 250(thymol and carvacrol)". With which results, you claimed this in discussion ? please clarify the subject? Or refer to your another published work?

## Response 7

- Corrected (Lines 77, 294, 295, 322, & 328)
